# Emoji use in social media posts: relationships with personality traits and word usage

**DOI:** 10.3389/fpsyg.2024.1343022

**Published:** 2024-02-05

**Authors:** Shelia M. Kennison, Kameryn Fritz, Maria Andrea Hurtado Morales, Eric Chan-Tin

**Affiliations:** ^1^Department of Psychology, Oklahoma State University, Stillwater, OK, United States; ^2^Department of Computer Science, Loyola University Chicago, Chicago, IL, United States

**Keywords:** social media, X (formerly Twitter), emojis, LIWC, personality traits, openness to experience, *you* pronouns

## Abstract

Prior research has demonstrated relationships between personality traits of social media users and the language used in their posts. Few studies have examined whether there are relationships between personality traits of users and how they use emojis in their social media posts. Emojis are digital pictographs used to express ideas and emotions. There are thousands of emojis, which depict faces with expressions, objects, animals, and activities. We conducted a study with two samples (*n* = 76 and *n* = 245) in which we examined how emoji use on X (formerly Twitter) related to users’ personality traits and language use in posts. Personality traits were assessed from participants in an online survey. With participants’ consent, we analyzed word usage in posts. Word frequencies were calculated using the Linguistic Inquiry Word Count (LIWC). In both samples, the results showed that those who used the most emojis had the lowest levels of openness to experience. Emoji use was unrelated to the other personality traits. In sample 1, emoji use was also related to use of words related to family, positive emotion, and sadness and less frequent use of articles and words related to insight. In sample 2, more frequent use of emojis in posts was related to more frequent use of *you* pronouns, *I* pronouns, and more frequent use of negative function words and words related to time. The results support the view that social media users’ characteristics may be gleaned from the content of their social media posts.

## 1 Introduction

Emojis (e.g., ☻ and ♥) are digital pictographs used to express ideas, frequently those conveying emotion (Danesi, 2016; Evans, 2017; Pardes, 2018; Bai et al., 2019). There are thousands of emojis with new emojis being created and used each year (Evans, 2017). Some emojis depict faces with expressions, animals, objects, and humans performing actions. They are used in a variety of contexts, including personal text messages and social media posts. Prior research has examined how emojis are used in communication (Saucier, 1994; Derks et al., 2008; Kaye et al., 2016, 2017; Pohl et al., 2017; Butterworth et al., 2019; Boutet et al., 2021) and how emoji use varies across different types of people (See Bai et al., 2019 for review). The focus of the present research was to investigate how emoji used on X (formerly Twitter) relates to users’ personality traits and the language used in posts.

Emojis were invented in 1999 and evolved from emoticons [e.g.,: -), ;-), and: @] (Ruan, 2011; Lee, 2018). The inventor of the emoji Shigetaka Kurita coined the word emoji from the Japanese words “e” (picture) and “moji” (character; Heisig, 2011). Studies have explored how emojis are used to convey information during communication (Derks et al., 2008; Novak et al., 2015; Ljubešić and Fišer, 2016; Pohl et al., 2017; Holtgraves and Robinson, 2020). Pohl et al. (2017) described multiple ways in which emojis may convey information. Emojis may be used to increase or decrease the emotional intent of a statement, as a reaction to a statement, as a standalone comment, in place of a word, or used as a flourish, which conveys little or no new information. Although emojis are becoming more frequently used (Evans, 2017), research has shown that users’ intended meaning of emojis is not always understood by viewers. Miller et al. (2016) asked participants to judge the meaning of a statement containing emojis (i.e., positive, negative, or neutral). They found that participants disagreed about the meaning of emojis 25% of the time. Recent research has also shown that the meaning of emojis can change over time (Robertson et al., 2021).

In the present research, we reasoned that our use of emojis during communication may relate to our personal characteristics. Increasingly, companies may be able to estimate the characteristics of current or prospective employees or customers through the analysis of social media posts using machine learning models (e.g., Xue et al., 2017; Receptiviti, 2023). Such approaches have been motivated by numerous studies demonstrating that the language we use holds clues to our personal characteristics (See Pennebaker, 2013 for review). Studies have demonstrated that language used on social media can be used to estimate to users’ age (Schwartz et al., 2013), gender (Schwartz et al., 2013; Chen et al., 2018; López-Rúa, 2021), and personality traits (Golbeck et al., 2011; Qiu et al., 2012; Hall and Pennington, 2013; Schwartz et al., 2013; Park et al., 2015; Marengo et al., 2017; Azucar et al., 2018; Li et al., 2019).

Among the earliest studies analyzing the content of posts, Golbeck et al. (2011) asked participants to complete a questionnaire that assessed the Big Five personality traits (i.e., extraversion, agreeableness, conscientiousness, openness to experience, and neuroticism). Prior research suggests that these traits have biological and environmental determinants and are relatively stable across the lifespan (McCrae and Costa, 1987; Digman, 1990; Costa and McCrae, 1992; Widiger, 2017). Golbeck et al. (2011) found that participants higher in extraversion used social words and words related to family more often. Participants higher in neuroticism (i.e., sometimes referred to as mood instability) used words related to perceptual processes (e.g., hearing, seeing, etc.) and words related to religion more often. Participants who had higher levels of openness to experience used words related to certainty and causation more often.

Few studies have examined the extent to which use of emoticons or emojis in social media posts relate to individual differences in personal characteristics (Hall and Pennington, 2013; Pohl et al., 2017; Li et al., 2018; López-Rúa, 2021; Aljasir, 2023). In a study of Facebook posts, Hall and Pennington (2013) assessed self-monitoring and Big Five personality traits and explored how they related to characteristics of posts. They found that people who use emoticons frequently were higher in self-monitoring and extraversion than those who used emoticons less often.

In another prior study, Marengo et al. (2017) assessed Big Five personality traits (i.e., extraversion, agreeableness, conscientiousness, openness to experience, and neuroticism) for English-speaking participants recruited through the Internet by researchers in Italy and Sweden. Participants rated 91 emojis from Apple’s Color Emoji font set (Apple, 2023), which had been pre-tested to be perceived as having some relationship with personality. Participants judged how well they recognized themselves in each emoji. The results showed that 36 out of 91 emojis were related to three personality traits (i.e., agreeableness, extraversion, and neuroticism/mood instability). Participants higher in extraversion rated emojis conveying positive meaning as more like them. Participants higher in agreeableness rated blushing face emojis as more like them. Participants higher in neuroticism/mood instability rated negative emojis as more like them.

Most recently, Li et al. (2018) examined how emoji use in posts was related to personality traits in a sample of posts. Using a machine learning model, they estimated users’ Big Five personality traits from frequencies of words used in the posts using the Linguistic Inquiry Word Count (LIWC; Pennebaker et al., 2015), which calculates frequencies for word categories (e.g., pronouns and other function words, words related to social relationships, emotion, biological concepts, etc.) These categories were established with research analyzing samples of text and speech from a wide variety of sources. In an early study using the LIWC, Pennebaker and King (1999) demonstrated that those with higher levels of neuroticism (or emotional stability) used the pronoun *I* and negative emotion words more often than others. Those with higher levels of extraversion used positive emotion words more frequently than those with lower levels of extraversion. Those with higher levels of openness used words over six letters more often than those with lower levels of openness. Li et al. (2018) used a machine learning algorithm trained with the LIWC word categories to estimate personality traits and showed that overall, emojis were used more frequently by users categorized as low in extraversion, high in agreeableness, and high in neuroticism. They also found that users categorized as high in agreeableness were more likely to use heart-shaped emojis and less likely to use negative emojis than other users. In analyses of positive and negative emojis, they found that users high in extraversion or higher in conscientiousness used positive emojis more often and negative emojis less often than other users. Users high in neuroticism used emojis with exaggerated facial expressions more than other users.

## 2 The study

The present research is among the first to examine how emoji use on X (formerly Twitter) is related to users’ self-reported Big Five personality traits and their language use in posts. We tested the hypothesis that individuals higher in agreeableness and mood instability and lower in extraversion may use emojis more frequently than others (c.f., Li et al., 2018). We also tested the hypothesis that emoji use would be related to word usage frequencies as assessed with LIWC (Pennebaker et al., 2015; See also Kacewicz et al., 2014), with categories related to emotion being related to emojis, particularly those most directly related to emotion (e.g., tone, positive emotion, negative emotion, affect, as well as others). We tested these hypotheses in two samples. In sample 1, we assessed the Big Five personality traits using Saucier’s (1994) mini-markers questionnaire. In sample 1, we assessed the personality trait of honesty-humility in addition to the Big Five personality traits using Ashton and Lee (2009) 60-item HEXACO questionnaire. Honesty-humility has been shown to be associated with sincerity, fairness, and genuineness. Prior research has demonstrated that the HEXACO provides comparably sound assessment of the openness to experience, extraversion, conscientiousness, neuroticism, and agreeableness (Lee and Ashton, 2004). We also explored the possibility that emoji use would be related to the honesty-humility trait, as one’s intention behind the use of emojis in social media posts may be to clarify the emotional intent of a verbal statement.

### 2.1 Methodology and procedure

After receiving approval for the research from the IRB, we recruited volunteers from a SONA system in a department of psychology. The data for sample 1 were collected during the 2020–2021 academic year. The data for sample 2 were collected during the 2021–2022 academic year. We used a correlational design for the study. We created our online survey using a professional license for Qualtrics. In the survey, participants provided information about their personality traits, gender, age in years, and their username for their X (formerly Twitter) public account. Participants could leave the response blank if they did not wish for their posts to be analyzed. We retrieved posts using the Twitter API with an academic research license. A script written in Python was used to retrieve posts. We were limited to downloading 3,200 posts per account; thus, the age of posts could differ across participants. A script written in Java was used to separate emojis used in posts for each user. For each participant, we computed the mean number of emojis used per post and the mean number of unique emojis used across all posts. We analyzed words appearing in posts using Pennebaker et al. (2015) LIWC 2015 application (Version 1.60 June 26, 2019). We used IBM SPSS Statistic 28 to analyze the data. The authors pledge to provide data, analytic methods, and study materials to other researchers upon request.

#### 2.1.1 Participants

In sample 1, there were 309 undergraduates (105 men, 200 women, 3 non-binary, 1 did not respond) who completed the online survey who received course credit in exchange for participation. Of these, 76 (52 women, 22 men, 2 other) volunteered to provide access to their posts from X (formerly Twitter) for the research. These participants were on average 24 years old (*SD* = 11.36). The sample was majority White (71%). The remainder of the sample identified as Native American (4%), Black/African American (9%), Hispanic (5%), or belonging to more than one group (11%). In sample 2, there were 577 undergraduates (153 men, 415 women, 5 non-binary, 2 other, 2 did not respond) who completed the online survey and received course credit in exchange for participation. Of these, 245 (67 men, 177 women, 1 other) volunteered to provide access to their posts from X (formerly Twitter) for the research. These participants were on average 20.10 years old (*SD* = 2.26). The sample included the following groups: White (76%), Native American (7%), Black/African American (5%), Hispanic (3%), and belonging to more than one group (9%).

#### 2.1.2 Materials

Participants completed surveys assessing sensation-seeking and general risk-taking behaviors, which have been reported in a prior publication (blinded for review, 2023, under review). In sample 1, participants also completed questions in which Big Five personality traits were measured. We assessed Big Five personality traits using Saucier’s (1994) 40-item mini marker questionnaire in which there are 8 adjectives to assess each trait (i.e., agreeableness, extraversion, conscientiousness, emotional instability/neuroticism, and openness to experience). Participants were asked to judge how accurate each adjective described them using a 9-point scale (i.e., *1 = extremely inaccurate, 2 = very inaccurate, 3 = moderately inaccurate; 4 = slightly inaccurate; 5 = neither accurate nor inaccurate; 6 = slightly accurate; 7 = moderately accurate; 8 = very accurate,* and *9 = extremely accurate*). Mean ratings were computed for each participant, after reverse scoring some items. Higher means reflect higher levels of each trait. The measure has been shown to have internal consistency (i.e., Cronbach alphas between 0.76 and 0.87, Kennison et al., 2021). We also found the measure to have high internal consistency in the present study (Cronbach alphas between α = 0.79 and α =0.90).

We used the following item to ask participants to consent to having their social media posts analyzed:

One aspect of this project is to determine how participants' personal characteristics are related to their use of language (word and phrase frequency) on social media platforms. Would you be okay with our collecting your publicly available information from your social media networks? Please enter the username for Twitter that you allow us to access.

In sample 2, we assessed the Big Five traits (i.e., extraversion, agreeableness, conscientiousness, emotionality/mood instability, and openness to experience) using the 60-item HEXACO-Revised (Ashton and Lee, 2009), which also assessed a sixth trait: honesty-humility. There were 10 items for each trait (e.g., *In social situations, I’m usually the one who makes the first move*). Participants rated items using a 5-point scale (*1 = strongly disagree, 5 = strongly agree*). After reverse scoring many items, each set of 10 items were averaged. Higher means reflect higher levels of the trait. Prior research has shown that the measure has good internal consistency. Cronbach alphas ranged from α = 0.73 to α = 0.80 (Ashton and Lee, 2009). In the present study, we also observed good internal consistency for the Big Five traits with Cronbach alphas ranging from α = 0.74 to α =0.79 and honesty-humility α = 0.67. We used a longer and more detailed paragraph in which to ask participants to provide their X (formerly Twitter) username:

One aspect of this project is to determine how participants' personal characteristics are related to their use of language (word and phrase frequency).

Researchers analyze the frequencies with which particular words and emojis are used on twitter. We would like to carry out similar analyses. We set up this survey to study these things anonymously. We are using two surveys with two separate databases to store your responses, so that the questions about your personality and other traits cannot be connected to your twitter account information. We are committed to protecting your anonymity.

### 2.2 Results

The total count for all emojis used across all user’s accounts was 16,574 in sample 1 and 58,139 in sample 2. The average emojis per account was 214.76 in sample 1 and 245.31 in sample 2. There were two participants who did not use any emojis in sample 1 and six in sample 2. We computed descriptive statistics and correlations for the mean number of emojis per post, number of unique emojis across posts, and the personality traits.

#### 2.2.1 Personality traits

We tested the hypothesis that there would be relationships between emoji use and personality by examining the correlations. The results from both samples supported the hypothesis. Tables 1, 2 displays the results for samples 1 and 2, respectively.

**Table 1:** Correlations and descriptive statistics for big five personality traits and emoji use on X (formerly Twitter) in sample 1.

		1	2	3	4	5	6	7	Mean	SD
1.	Mean Emoji Use		0.53^***^	0.09	−0.18	−0.10	−0.10	−0.33^**^	0.43	0.34
2.	Mean Unique Emojis			0.02	−0.18	−0.12	−0.18	−0.26^*^	46.80	45.82
3.	Extraversion				0.43^***^	0.40^***^	0.50^***^	0.35^**^	4.76	1.69
4.	Agreeableness					0.70^***^	0.68^***^	0.76^***^	6.19	1.81
5.	Conscientiousness						0.57^***^	0.69^***^	5.83	1.70
6.	Mood Instability							0.58^***^	5.11	1.94
7.	Openness								6.01	1.60

*p < 0.05, **p < 0.01, ***p < 0.001.

**Table 2:** Correlations and descriptive statistics HEXACO personality traits and emoji use on X (formerly Twitter) in sample 2.

		1	2	3	4	5	6	7	8	Mean	SD
1.	Mean Emoji Use		0.64^***^	−0.13^*^	0.05	−0.004	0.10	0.07	−0.03	0.35	0.34
2.	Mean Unique Emojis			−0.11	−0.04	−0.07	0.06	0.02	−0.12	0.09	. 10
3.	Openness				−0.08	0.06	−0.03	0.10	0.09	3.20	0.67
4.	Extraversion					0.17^**^	0.09	−0.16^*^	−0.05	3.33	0.67
5.	Agreeableness						0.13^*^	0.07	0.26^***^	3.23	0.61
6.	Conscientiousness							0.12	0.26^***^	3.66	0.64
7.	Emotionality								0.07	3.55	0.68
8.	Honesty/Humility									3.25	0.58

*p < 0.05, **p < 0.01, ***p < 0.001.

In sample 1, more frequent emoji use was associated with lower levels of openness were related to using more emojis (*r* = −0.33, *p* = 0.003) and using a wider variety of emojis (*r* = −0.26, *p* = 0.022). Emoji use was unrelated to the other Big Five traits (i.e., extraversion, agreeableness, conscientiousness, and mood instability). We explored further how Big Five traits were related to emoji use by conducting a hierarchical multiple regression in which mean emojis per post was the dependent variable and gender was entered as block 1, openness to experience in Block 2, and the remaining four Big Five traits were entered in Block 3 to determine whether any additional variance could be explained after considering gender and openness. In all the regression analyses reported in this paper, we confirmed that the assumptions were met (Field, 2013). The results showed that in Block 1, gender was significant, accounting for 4% of the variance in emoji use. Women used more emojis than men (*β* = −0.23, *p* = 0.049): *F*(1, 75) = 4.00, *p* = 0.049. In Block 2, openness to experience was significant, *F*(2, 75) = 5.45, *p* = 0.006, accounting for 8% additional variance in emoji use (*β* = −0.29, *p* = 0.012). The change in *R^2^* was significant, *F*(1, 73) =6.59, *p* < 0.001. Those reporting lower levels of openness use the most emojis. Block 3, which included the remaining Big Five personality traits, was significant: *F*(6,75) = 2.58, *p* = 0.026; however, the change in *R^2^* was not significant, *F*(4, 69) =1.13, *p* = 0.349, indicating that no additional variance in emoji use was accounted for by the extraversion, agreeableness, conscientiousness, and emotional instability. Table 3 provides a summary of these results.

**Table 3:** Summary of hierarchical regression predicting mean emojis per post and mean unique emojis per post from sample 1.

	Mean emojis per post				Mean unique emojis per post			
Predictor	*β*	*t*	R^2^	ΔR^2^	*β*	*t*	R^2^	ΔR^2^
Block 1			0.05	0.05			0.02	0.03
Gender	0.23	2.00^*^			0.18	1.58		
Block 2			0.109	0.079^*^			0.11	0.10^**^
Gender	0.14	5.54^***^			0.09	0.76		
Openness	−0.29	−2.57^***^			−0.32	−2.82^*^		
Block 3			0.11	0.054			0.08	0.03
Gender	0.08	0.64			0.06	0.45		
Openness	−0.49	−2.50^*^			−0.40	−1.99^*^		
Extraversion	0.20	1.53			0.19	1.43		
Agreeableness	−0.01	−0.04			−0.01	−0.04		
Conscientiousness	0.15	0.88			0.03	0.17		
Mood Instability	0.02	0.11			−0.02	−0.15		

*p < 0.05, **p < 0.01, ***p < 0.001.

We found similar results when examining mean unique emojis per post. We carried out a hierarchical multiple regression in which mean unique emojis per post was the dependent variable and the same predictor variables were into blocks in the same manner: Block 1 (gender), Block 2 (openness), and Block 3 (the remaining personality traits). The results showed that in Block 1, gender was not significant: *F*(1, 74) = 2.51, *p* = 0.117. In Block 2, openness to experience was significant, *F*(2, 74) = 5.35, *p* = 0.007, accounting for 9.6% additional variance in emoji use (*β* = −0.40, *p* = 0.05). The change in *R^2^* was significant, *F*(1, 72) = 7.94, *p* = 0.006, suggesting that those reporting lower levels of openness to experience create posts with a greater variety of emojis than other users. Block 3, which included the remaining Big Five personality traits, was not significant: *F*(6, 74) = 2.13, *p* = 0.06. The change in *R^2^* was not significant, *F*(4, 68) = 0.58, *p* = 0.67, indicating that no additional variance in emoji use. A summary of these results is also displayed in Table 3.

The results for sample 2 were similar to those found for sample 1. More frequent emoji use was associated with lower levels of openness to experience were related to mean emojis used per post (*r* = −0.13, *p* = 0.04). There were no other significant results involving the other Big Five personality traits and use of emojis. We further examined how Big Five traits were related to emoji use by conducting a hierarchical multiple regression in which mean emojis per post was the dependent variable and gender was entered as block 1, openness to experience in Block 2, and the remaining five HEXACO traits were entered in Block 3 to determine whether any additional variance could be explained after considering gender and openness to experience. In the analysis predicting mean emojis per post, the results showed that in Block 1, gender was significant, accounting for 4% of the variance in emoji use. Women used more emojis than men (*β* = −0.17, *p* = 0.011): *F*(1, 233) = 6.64, *p* = 0.011. In Block 2, openness to experience was significant, *F*(2, 234) = 5.31, *p* = 0.006, accounting for 2% additional variance in emoji use. The change in *R^2^* was significant, *F*(1, 232) = 3.90, *p* = 0.049. As in sample 1, we found that those reporting lower levels of openness to experience use the most emojis (*β* = −0.13, *p* = 0.049). Block 3, which included the remaining HEXACO personality traits, was not significant: *F*(7,234) = 1.83, *p* = 0.083. The change in *R^2^* was not significant, *F*(5, 227) = 0.46, *p* = 0.81, indicating that no additional variance in emoji use was accounted for by the extraversion, agreeableness, conscientiousness, emotionality/mood instability, and honesty-humility. Table 4 displays a summary of these results.

**Table 4:** Summary of hierarchical regression predicting mean emojis per post and mean unique emojis per post from sample 2.

	Mean emojis per post				Mean unique emojis per post			
Predictor	*β*	*t*	*R*^2^	Δ*R*^2^	*β*	*t*	R^2^	ΔR^2^
Block 1			0.02	0.03^*^			0.02	0.03^*^
Gender	0.17	2.58^*^			0.17	2.58^*^		
Block 2			0.04	0.03^*^			0.04	0.02^*^
Gender	0.16	2.47^**^			0.15	2.04		
Openness	−0.13	1.98^*^			−0.12	−1.82^*^		
Block 3			0.02	0.01			0.02	0.01
Gender	0.15	2.04			0.15	2.04		
Openness	−0.12	−1.82^*^			−0.12	−1.82^*^		
Extraversion	0.03	0.37			0.03	0.37		
Agreeableness	0.01	0.12			0.01	0.12		
Conscientiousness	0.09	1.27			0.09	1.27		
Mood Instability	0.01	0.07			0.01	0.17		
Honesty	−0.06	−0.86			−0.06	−0.86		

*p < 0.05, **p < 0.01, ***p < 0.001.

We also conducted a hierarchical multiple regression in which mean number of unique emojis per post was the dependent variable and the same three blocks of predictor variables: gender in Block 1, openness to experience in Block 2, and the remaining HEXACO traits in Block 3. In the analysis predicting mean number of unique emojis per post, the results showed that in Block 1, gender was significant, accounting for 3% of the variance in unique emoji use. Women used more emojis than men (*β* = 0.16, *p* = 0.016): *F*(1, 234) = 5.91, *p* = 0.016. In Block 2, openness to experience was significant, *F*(2, 234) = 4.48, *p* = 0.012; however, the change in *R^2^* was not significant, *F*(1, 232) = 3.00, *p* = 0.085. Block 3, which included the remaining HEXACO personality traits, was significant: *F*(7,234) = 2.39, *p* = 0.022; however, the change in *R^2^* was not significant, *F*(5, 227) = 1.53, *p* = 0.18, indicating that no additional variance in emoji use was accounted for by the remaining: extraversion, agreeableness, conscientiousness, emotionality, and honesty-humility. Table 4 displays a summary of these results.

#### 2.2.2 LIWC analyses of word usage

To test the hypothesis that emoji use would be related to language use in posts, we conducted correlations between mean number of emojis per post, number of unique emojis across posts, and the LIWC categories (See Pennebaker et al., 2015). The results from both samples supported the hypothesis. Table 5 displays the significant correlations between LIWC words categories and mean emojis used per post for sample 1. To explore further the relationships among emoji use and words usage frequencies, we carried out a multiple regression in which mean emojis per post were used as the dependent variable. We entered six word usage categories, which were subordinate LIWC word categories, with the strongest relationships with mean emojis (i.e., *p < 0.*01) and gender as independent variables simultaneously. Only two variables were not significant predictors (i.e., gender and sexual words). After removing those variables, we observed a significant model accounting for 47% of the variance (adjusted *R^2^* = 0.472), *F*(5, 75) = 14.41, *p* < 0.001. There were five significant predictors: family (*β* = 0.34, *p* < 0.007), sad (*β* = 0.25, *p* = 0.01), insight (*β* = −0.257, *p* = 0.02), positive emotion (*β* = 0.26, *p* = 0.009), articles (*β* = −0.309, *p* = 0.004). A summary of the results is provided in Table 6. We also explored the relationships between the mean number of unique emojis used per post and word usage frequencies. These results are displayed in Table 7. We entered the word usage categories, which were subordinate LIWC word categories, with the strongest relationships with mean unique emojis (i.e., *p < 0.*01) and gender as independent variables. The model was not significant: *F*(3, 75) = 1.00, *p* = 0.398.

**Table 5:** Correlations for mean emojis per post and LIWC word categories from sample 1.

Word category	Mean emoji per post
Family	0.39^***^
Positive emotion	0.34^**^
Sad	0.34^**^
Feel	0.28^*^
*You* pronouns	0.26^*^
Body	0.27^*^
Article	−0.36^**^
Insight	−0.36^**^
Money	−0.29^*^
Anger	−0.26^*^
Sexual	−0.34^**^
Ingest	−0.25^*^
Risk	−0.25^*^
Swear words	−0.24^*^

*p < 0.05, **p < 0.01, ***p < 0.001.

**Table 6:** Summary of multiple regression analyses predicting mean emojis per X post (formerly Post) in sample 1.

	Mean emojis per post	
Predictor	*β*	*t*
Family	0.34	3.99^***^
Positive emotion	0.26	2.67^**^
Sad	0.25	2.64^*^
Articles	−0.31	−2.97^**^
Insight	−0.26	−2.48^*^

^*^p < 0.05, ^**^p < 0.01, ^***^p < 0.001, β, standardized coefficient beta.

**Table 7:** Correlations for mean unique emojis per post and LIWC word categories from sample 1.

Word category	Mean unique emojis per post
Body	0.38^***^
Sad	0.35^**^
Females	0.35^**^
Family	0.28^*^
Insight	−0.25^*^
Words longer than six letters	−0.28^*^
Positive emotion	0.25^*^

*p < 0.05, **p < 0.01, ***p < 0.001.

For sample 2, we also observed a higher number of significant results between LIWC word categories and mean emojis used per post than we did for sample 1. These results are displayed in Table 8. To explore further the relationships among emoji use and words usage frequencies, we carried out a multiple regression in which mean emojis per post were used as the dependent variable. We entered 19 word usage categories, which were subordinate LIWC word categories, with the strongest relationships with mean emojis per post (i.e., *p < 0.*01) and gender as independent variables simultaneously. Twelve variables were not significant predictors. After removing the non-significant predictor variables from the analysis, we observed a significant model. A summary of the results is provided in Table 9. The model accounted for 32% of the variance (i.e., adjusted *R^2^* = 0.31.8), *F*(7,234) = 16.61, *p* < 0.001. All seven of the predictor variables were significant: *you* pronouns (*β* = 0.35, *p* < 0.001), I (β = 0.20, *p* = 0.013), adjectives (*β* = 0.29, *p* < 0.001), negations (β = 0.32, *p* < 0.001), time (*β* = 0.31, *p* < 0.001), number (β = −0.39, *p* < 0.001), and dictionary words (*β* = −0.85, *p* < 0.001).

**Table 8:** Correlations for mean emojis per post and LIWC word categories from sample 2.

Word category	Mean emoji per post
*You* pronouns	0.44^***^
Adjective	0.37^***^
Focus present	0.33^***^
Focus future	0.31^***^
Dic	0.29^***^
Verb	0.28^***^
Negate	0.27^***^
Auxverb	0.27^***^
Netspeak	0.27^**^
Adverb	0.24^***^
Compare	0.23^***^
Friend	0.20^**^
Reward	0.20^**^
Time	0.24^***^
I	0.19^**^
sad	0.18^**^
family	0.17^*^
quant	0.16^*^
positive emotion	0.15^*^
negative emotion	0.15^*^
female	0.15^*^
Discrepancy	0.14^*^
differ	0.14^*^
see	0.14^*^
leisure	0.14^*^
religion	0.18^**^
number	−0.34^***^
WPS	−0.24^***^

^*^p < 0.05, ^**^p < 0.01, ^***^p < 0.001.

**Table 9:** Summary of multiple regression analyses predicting mean emojis per X post (formerly Post) in sample 2.

Predictor variable	*β*	*t*
Dictionary words	−0.85	5.61^***^
Number	−0.39	−4.39^***^
You pronouns	0.35	5.61^***^
Negate	0.32	4.15^***^
Time	0.31	4.04^***^
Adjectives	0.29	3.87^***^
I	0.20	2.51^*^

^*^p < 0.05, ^**^p < 0.01, ^***^p < 0.001, β, standardized coefficient beta.

Using a wider variety of emojis was also found to be related to LIWC word categories. Table 10 displays these results. To explore further how word usage might be useful in predicting use of a greater variety of emojis, we conducted a multiple regression in which mean unique emojis per post was the dependent variable. Gender and the 14 subordinate LIWC categories with the strongest relationships with mean unique emojis per post (i.e., *p < 0.*01) and gender as independent variables simultaneously as predictor variables. After removing the non-significant predictor variables from the analysis, we observed a significant model. A summary of the results is provided in Table 11. The model accounted for 16% of the variance (i.e., adjusted R^2^ = 0.16), *F*(4, 234) = 12.38, p < 0.001. All four predictor variables were significant: *you* pronouns (*β* = 0.29, *p* < 0.001) and words related to seeing (*β* = 0.16, *p* = 0.009), leisure (*β* = 0.17, *p* = 0.005), and religion (*β* = 0.13, *p* = 0.045).

**Table 10:** Correlations for mean unique emojis per post and LIWC word categories from sample 2.

Word category	Mean unique emoji per post
You	0.31^***^
Positive emotion	0.31^**^
Adjective	0.24^***^
Netspeak	0.24^***^
Compare	0.23^***^
Focus present	0.22^**^
See	0.20^**^
Hear	0.20^**^
Friend	0.19^**^
Dictionary words	0.19^**^
Leisure	0.18^**^
Religion	0.18^**^
Verb	0.16^*^
Reward	0.15^*^
Adverb	0.14^*^
Negate	0.14^*^
We	0.13^*^
Number	−0.29^***^
Words per sentence	−0.27^***^
Death	−0.14

^*^p < 0.05, ^**^p < 0.01, ^***^p < 0.001.

**Table 11:** Summary of multiple regression analyses predicting number of unique emojis in sample 2.

Predictor variable	*β*	*t*
you pronouns	0.29	4.75^***^
see	0.16	2.62^**^
leisure	0.17	2.81^**^
religion	0.13	2.02^*^

^*^p < 0.05, ^**^p < 0.01, ^***^p < 0.001. β, standardized coefficient beta.

## 3 General discussion

The present research examined emoji use on X (formerly Twitter) and whether personality traits and word usage were related to the frequency of emoji use in posts. We reported results from two samples, varying in size. In both samples, we observed that participants reporting lower levels of openness to experience used emojis more often and also used a wider variety of emojis. There were relationships between emoji use and some of the LIWC word categories. The LIWC categories differed for the two samples. In sample 1, more frequent use of emojis in posts was related to more frequent use of words related to family, positive emotion, and sadness. Less frequent use of emojis was related to more frequent use of articles and words related to insight. In sample 2, the larger of the two samples, more frequent use of emojis was related to more frequent use of *you*-pronouns, *I*-pronouns, adjectives, negative function words (e.g., *no, not, never*), and words related to time. More frequent use of emojis was also related to using fewer dictionary words and numbers. Those using a larger variety of emojis also used *you* pronouns and words related to seeing, leisure, and religion more frequently than those using a smaller variety of emojis.

The present results contrast with the few prior studies examining personality and emoticon or emoji use in social media posts (Hall and Pennington, 2013; Li et al., 2018). Hall and Pennington (2013) found users higher in extroversion used emoticons more frequently on Facebook than those lower in extroversion. We did not observe any relationships involving extroversion and emoji use. It is worth noting that in the decade since Hall and Pennington (2013) study, the use of social media and the use of emojis has increased substantially (Evans, 2017). Over time, patterns in emoji use may have changed. In recent research, Robertson et al. (2021) documented that the meaning of some emojis has changed between 2012 and 2018. It is unclear whether meaning changes for emojis over time may also lead to changes in the relationships between users’ characteristics and their use of specific emojis. We suspect that the typical user of emojis in 2013 could differ in many ways, including personality, from the typical user of emojis today.

Our results also differ from those reported by Li et al. (2018). They found links between emoji use and extraversion, agreeableness, and conscientiousness using machine learning to estimate users’ Big Five personality traits. We did not observe relationships involving extraversion, agreeableness or conscientiousness. The difference in the present results and those from Li et al. (2018) study may relate from the differences in how users’ Big Five personality traits were determined as well as differences in the population(s) represented in the sample. The present results were drawn from a population of undergraduates in the central region of the United States, which is one of its limitations. In Li et al. (2018), the sample reflects a more diverse population in terms of age and education. There are additional differences between the methodology used by Li et al. (2018) and the present study. Li et al. (2018) estimated users’ personality traits using word frequencies from their social media. In contrast, in the present study, we assessed personality traits directly from participants themselves. Other differences include the fact that Li et al. (2018) restricted their analysis to accounts with at least 500 posts. In the present study, we include accounts with as few as 100 posts. Li et al. (2018) also excluded from their dataset accounts of users whose mean emoji use per post was above 0.95 or below 0.05, which was not done for the present study.

The results are novel in that they are the first to demonstrate a relationship between emoji use and openness to experience. The findings merit future research into which of the multiple aspects of openness to experience may be most strongly related to emoji use. Prior research has suggested that each of the Big Five personality traits reflect multiple facets (Costa and McCrae, 1992). Openness to experience involves six facets: adventurousness, being imaginative, being intellectually curious, questioning authority, being emotionally aware, and being interested in the arts. Future research is needed to examine to what extent emoji use is related to one or more of the facets of openness. A more fine-grained analysis of openness to experience is needed to determine which facet(s) are most strongly related to emoji use.

The present study is also novel in that we also examined how emoji use was related to word usage in posts. We observed that different word frequency categories were related to emoji use in our two samples. Only one category of word frequency emerged as a significant predictor in more than one analysis. The frequency of *you* pronouns was related to using more emojis and also using a wider variety of emojis. Prior research has suggested that the use of *you* pronouns reflect a focus on others, rather than focus on self (Kacewicz et al., 2014). This is consistent with the users choosing to communicate with an emoji when they are focused on or communicating to others. In sample 2, the larger sample, we found that those using the most emojis used fewer dictionary words, which suggests that users may be using some emojis instead of words. Word replacement is just one possible use of an emoji (Evans, 2017). It is somewhat surprising that our results found only hints that emoji use was related to communicating emotion. In sample 1, more frequent emoji use was related to more frequent use of words related to positive emotion and sadness. These relationships were not observed in the larger sample where more frequent emoji use was related to more frequent use of negative function words (e.g., *no, not, never*). Future research is needed to explore this relationship further. We speculate that it may reflect a communication strategy in which one uses emojis to soften the emotional impact of a negative sentiment, as in *I would never go back to that restaurant* or You’re never getting me to go on a blind date again. This line of research is challenging as it will require close examination of the context in which emojis are used. It will also require that researchers make judgments about the users’ intentions or require researchers to ask participants to provide information about their intentions in posts in retrospect or during the composition of posts.

The present research has multiple limitations. For both samples, we observed a reluctance on the part of participants to opt in to have their social media posts analyzed. In sample 1, only about 25 percent of participants provided their X (formerly Twitter) username. In sample 2, after we improved the way that we invited participants to opt in, 42 percent of participants provided their username. Future studies are needed to determine whether the present results generalize to other samples of social media posts on X as well as other platforms. A second important limitation in our study is that samples were drawn from college students, whose use of emojis may differ from that of other populations. Future research is needed to determine whether the present results generalize to other types of adults, such as those without college experience or from different cultural backgrounds (See Aljasir, 2023 for discussion of cultural differences in emoji use). Our samples were also majority female. We found that women used emojis more often than men, a finding that has been documented in prior research (López-Rúa, 2021). Future research on the different motivations for using emojis is needed to explore the relationship between gender and emoji use. Lastly, our results were obtained from analyses of posts on X (formerly Twitter). It is possible that the social norms in emoji use differ across social media platforms. Furthermore, different social media platforms may attract different types of users. Future research is needed to examine whether users’ emoji use differs across different social media platforms.

Applications of these results include analyzing emoji use of prospective employees or customers in industries in which openness to experience is important (e.g., entertainment and scientific research). Frequent use of emojis and using a wider variety of emojis could be indicative of one of more of the following: lower levels of imagination, adventurousness, curiosity, emotional awareness, interests in the arts, and/or questioning of authority. Future research is needed to determine whether emoji use is related to each of the facets of openness to experience or only a subset of these facets.

## Data availability statement

The raw data supporting the conclusions of this article will be made available by the authors, without undue reservation.

## Ethics statement

The studies involving humans were approved by Oklahoma State University Stillwater IRB. The studies were conducted in accordance with the local legislation and institutional requirements. Written informed consent was not required to participate in this study in accordance with the local legislation and institutional requirements.

## Author contributions

SK: Conceptualization, Data curation, Formal analysis, Funding acquisition, Investigation, Methodology, Project administration, Writing – original draft, Writing – review & editing. KF: Data curation, Software, Visualization, Writing – original draft. MM: Data curation, Investigation, Writing – original draft, Conceptualization. EC-T: Conceptualization, Funding acquisition, Writing – review & editing.

## References

[ref1] Aljasir S. (2023). Emoji as a social presence tool among Arab digital media users: do the demographic variables of the sender play a role? Soc. Sci. Comput. Rev. doi: 10.1177/08944393231181638

[ref2] Apple. (2023). Emojipedia. Available at: https://emojipedia.org/apple/

[ref3] Ashton M. C. Lee K. (2009). The HEXACO-60: a short measure of the major dimensions of personality. J. Pers. Assess. 91, 340–345. doi: 10.1080/00223890902935878, PMID: 20017063

[ref4] Azucar D. Marengo D. Settanni M. (2018). Predicting the big 5 personality traits from digital footprints on social media: a meta-analysis. Personal. Individ. Differ. 124, 150–159. doi: 10.1016/j.paid.2017.12.018

[ref5] Bai Q. Dan Q. Mu Z. Yang M. (2019). A systematic review of emoji: current research and future perspectives. Front. Psychol. 10:2221. doi: 10.3389/fpsyg.2019.02221 31681068 PMC6803511

[ref6] Boutet I. LeBlanc M. Chamberland J. A. Collin C. A. (2021). Emojis influence emotional communication, social attributions, and information processing. Comput. Hum. Behav. 119:106722. doi: 10.1016/j.chb.2021.106722

[ref7] Butterworth S. E. Giuliano T. A. White J. Cantu L. Fraser K. C. (2019). Sender gender influences emoji interpretation in text messages. Front. Psychol. 10:784. doi: 10.3389/fpsyg.2019.00784 31024407 PMC6459937

[ref8] Chen Z. Lu X. Ai W. Li H. Mei Q. Liu X. (2018). Through a gender lens: learning usage patterns of emojis from large-scale android users. In *Proceedings of the 2018 World Wide Web Conference*, 763–772.

[ref9] Costa P. T. McCrae R. R. (1992). Neo Pi-R. Odessa, FL: Psychological Assessment Resources.

[ref10] Danesi M. (2016). The semiotics of emoji. US: Bloomsbury.

[ref11] Derks D. Bos A. E. Von Grumbkow J. (2008). Emoticons and online message interpretation. Soc. Sci. Comput. Rev. 26, 379–388. doi: 10.1177/0894439307311611, PMID: 22394421

[ref12] Digman J. M. (1990). Personality structure: emergence of the five-factor model. Annu. Rev. Psychol. 41, 417–440. doi: 10.1146/annurev.ps.41.020190.002221, PMID: 38014385

[ref13] Evans V. (2017). The emoji code: How smiley faces, love hearts and thumbs up are changing the way we communicate. New York: Picador.

[ref14] Field A. (2013). Discovering statistics using IBM SPSS statistics. New York: Sage.

[ref15] Golbeck J. Robles C. Edmondson M. Turner K. (2011). Predicting personality from twitter. 2011 *IEEE third international conference on privacy, security, risk and trust and 2011 IEEE third international conference on social computing*, 149–156.

[ref18] Hall J. A. Pennington N. (2013). Self-monitoring, honesty, and cue use on Facebook: the relationship with user extraversion and conscientiousness. Comput. Hum. Behav. 29, 1556–1564. doi: 10.1016/j.chb.2013.01.001

[ref19] Heisig J. W. (2011). Remembering the kanji 1: A complete course on how not to forget the meaning and writing of Japanese characters. Honolulu, HI: University of Hawaii Press.

[ref20] Holtgraves T. Robinson C. (2020). Emoji can facilitate recognition of conveyed indirect meaning. PLoS One 15:e0232361. doi: 10.1371/journal.pone.0232361, PMID: 32353045 PMC7192449

[ref21] Kacewicz E. Pennebaker J. W. Davis M. Jeon M. Graesser A. C. (2014). Pronoun use reflects standings in social hierarchies. J. Lang. Soc. Psychol. 33, 125–143. doi: 10.1177/0261927X13502654

[ref22] Kaye L. K. Malone S. A. Wall H. J. (2017). Emojis: insights, affordances, and possibilities for psychological science. Trends Cogn. Sci. 21, 66–68. doi: 10.1016/j.tics.2016.10.007, PMID: 28107838

[ref23] Kaye L. K. Wall H. J. Malone S. A. (2016). Turn that frown upside-down: a contextual account of emoticon usage on different virtual platforms. Comput. Hum. Behav. 60, 463–467. doi: 10.1016/j.chb.2016.02.088

[ref24] Kennison S. M. Jones I. T. Spooner V. H. Chan-Tin D. E. (2021). Who creates strong passwords when nudging fails? Computers in Human Behav. Reports. 4:100132. doi: 10.1016/j.chbr.2021.100132

[ref25] Lee S. (2018). Emoji at MoMA: considering the 'original emoji' as art. First Monday. 23:9. doi: 10.5210/fm.v23i9.9401

[ref26] Lee K. Ashton M. C. (2004). Psychometric properties of the HEXACO personality inventory. Multivar. Behav. Res. 39, 329–358. doi: 10.1207/s15327906mbr3902_8, PMID: 26804579

[ref27] Li W. Chen Y. Hu T. Luo J. (2018). Mining the relationship between emoji usage patterns and personality. In *Proceedings of the international AAAI conference on web and social media*. Vol. 12.

[ref28] Li M. Chng E. Chong A. Y. L. See S. (2019). An empirical analysis of emoji usage on twitter. Ind. Manag. Data Syst. 119, 1748–1763. doi: 10.1108/IMDS-01-2019-0001

[ref29] Ljubešić N. Fišer D. (2016). A global analysis of emoji usage. In *Proceedings of the 10th web as corpus workshop* (pp. 82–89).

[ref30] López-Rúa P. (2021). Men and women on twitter: a preliminary account of British emoji usage in terms of preferred topics and gender-related habits. Lang. Int. 19. Available at: https://www.languageatinternet.org/articles/2021

[ref31] Marengo D. Giannotta F. Settanni M. (2017). Assessing personality using emoji: an exploratory study. Pers. Individ. Differ. 112, 74–78. doi: 10.1016/j.paid.2017.02.037, PMID: 35185663

[ref32] McCrae R. R. Costa P. T. (1987). Validation of the five-factor model of personality across instruments and observers. J. Pers. Soc. Psychol. 52, 81–90. doi: 10.1037/0022-3514.52.1.81, PMID: 3820081

[ref33] Miller H. Thebault-Spieker J. Chang S. Johnson I. Terveen L. Hecht B. (2016). “Blissfully happy” or “ready to fight”: varying interpretations of emoji. In *Proceedings of the international AAAI conference on web and social media* (*Vol. 1*, pp. 259–268).

[ref34] Novak K. Smailović J. Sluban B. Mozetič I. (2015). Sentiment of Emojis. PLoS One 10:e0144296. doi: 10.1371/journal.pone.0144296, PMID: 26641093 PMC4671607

[ref35] Pardes Arielle (2018). The WIRED guide to emoji. Available at: https://www.wired.com/story/guide-emoji/

[ref36] Park G. Schwartz H. A. Eichstaedt J. C. Kern M. L. Kosinski M. Stillwell D.. (2015). Automatic personality assessment through social media language. J. Pers. Soc. Psychol. 108, 934–952. doi: 10.1037/pspp0000020, PMID: 25365036

[ref37] Pennebaker J. W. (2013). The secret life of pronouns: What our words say about us. US: Bloomsbury.

[ref38] Pennebaker J. W. Boyd R. L. Jordan K. Blackburn K. (2015). The development and psychometric properties of LIWC2015. US: University of Texas at Austin.

[ref39] Pennebaker J. King L. (1999). Linguistic styles: language use as an individual difference. J. Pers. Soc. Psychol. 77:1296. doi: 10.1037/0022-3514.77.6.1296, PMID: 10626371

[ref40] Pohl H. Domin C. Rohs M. (2017). Beyond just text: semantic emoji similarity modeling to support expressive communication. ACM Transac. Computer-Human Interaction (TOCHI) 24:6, 1–41. doi: 10.1145/3039685

[ref42] Qiu L. Lin H. Ramsay J. Yang F. (2012). You are what you post: personality expression and perception on twitter. J. Res. Pers. 46, 710–718. doi: 10.1016/j.jrp.2012.08.008

[ref43] Receptiviti. (2023). Talent assessment. Available at: https://www.receptiviti.com/talent-assessment

[ref44] Robertson A. Liza F. F. Nguyen D. McGillivray B. Hale S. A. (2021). Semantic journeys: quantifying change in emoji meaning from 2012-2018 (paper presentation). *15th International AAAI Conference*.

[ref45] Ruan L. (2011). Meaningful signs—emoticons. Theory and Practice in Lang. Stud. 1, 91–94. doi: 10.4304/tpls.1.1.91-94

[ref1002] Saucier G. (1994). Mini-markers: A brief version of goldberg’s unipolar big-five markers. J. Pers. Assess. 63, 506–516. doi: 10.1207/s15327752jpa6303_8 7844738

[ref46] Schwartz H. A. Eichstaedt J. C. Kern M. L. Dziurzynski L. Ramones S. M. Agrawal M.. (2013). Personality, gender, and age in the language of social media: the open-vocabulary approach. PLoS One 8:e73791. doi: 10.1371/journal.pone.0073791, PMID: 24086296 PMC3783449

[ref48] Widiger T. A. (Ed.). (2017). The Oxford handbook of the five factor model. Oxford: Oxford University Press.

[ref49] Xue D. Hong Z. Guo S. Liang L. Wu L. Zheng J.. (2017). Personality recognition on social media with label distribution learning. IEEE Access 5, 13478–13488. doi: 10.1109/ACCESS.2017.2719018, PMID: 35712070

